# Protocol for formation, staining, and imaging of 3D breast cancer models using MicroTissues mold systems

**DOI:** 10.1016/j.xpro.2024.103250

**Published:** 2024-09-20

**Authors:** Ben Haspels, Maayke M.P. Kuijten

**Affiliations:** 1 Department of Molecular Genetics, Erasmus MC, 3000 CA, Rotterdam, the Netherlands; 2 Oncode Institute, Erasmus MC Cancer Institute, Erasmus MC, 3015 GD, Rotterdam, the Netherlands

**Keywords:** cell biology, cancer, microscopy

## Abstract

The use of 3D cancer models to study therapy response has a great advantage over conventional 2D cell culture. Here, we present an optimized protocol for breast cancer spheroid and rod-shaped microtissue formation using MicroTissues mold systems. We describe steps for cast formation and treating the 3D models with DNA-damaging agents. We then detail procedures for analyzing the 3D models by whole-mount immunostaining and confocal imaging of fixed samples or with the use of live-cell reporters.

## Highlights

• Instructions for cast formation required to form spheroids and rods
• Procedure to form spheroids and rods and to treat these models
• Instructions for harvesting of 3D cell models and immunostaining of spheroids
• Instructions for confocal imaging of live and fixed 3D cell models

## Abstract (editor highlights)

Publisher’s note: Undertaking any experimental protocol requires adherence to local institutional guidelines for laboratory safety and ethics.

## Graphical abstract

**Figure:**
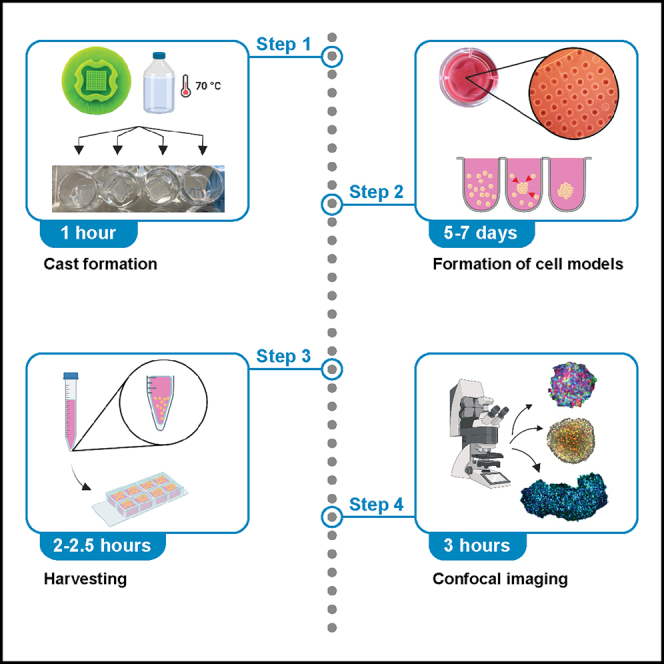

## Before you begin

Breast cancer is the most common type of cancer in women worldwide and approximately 20% of patients develop metastatic disease, which is very difficult to treat. This emphasizes the importance of studying treatment response in breast cancer in a physiologically relevant model. Use of 3D cell models has great advantages over conventional 2D cell culture, as 3D models are more physiologically relevant and better comparable to an *in vivo* tumor.^1^ This protocol describes the formation, harvesting, immunofluorescence staining and imaging of spheroids and rod-shaped micro-tissues using MCF7 and MDA-MB-436 breast cancer cells. In addition, we used MCF7 cells stably-transfected with several reporter plasmids to show the potential of this protocol without any staining procedures. For formation of 3D models we used commercially available molds (MicroTissues) in various shapes and sizes and initially used the manufacturer’s manual, which we further optimized and expanded for our applications.

***Note:*** Although we describe in this protocol the use of breast cancer cell lines MCF7 and MDA-MB-436 only, we have also successfully used these methods for A549 (lung cancer) and DU145 (prostate cancer) cell lines. Imaging of these 3D models was performed with a Leica TCS SP8 confocal microscope. General use of this microscope is not described in this protocol and can be found on the website of Leica Microsystems.

1. Perform all steps for formation and harvesting of 3D micro-tissues aseptically in a flow cabinet.
2. Prepare all materials and reagents in advance and make sure everything is sterilized when applicable.
3. Cells are cultured in a humidified incubator at 37°C with 5% CO_2_ injection (90%–95% humidity).

## Key resources table

**Table:** 

REAGENT or RESOURCE	SOURCE	IDENTIFIER
**Chemicals, peptides, and recombinant proteins**		

Penicillin-streptomycin (PS)	Sigma-Aldrich	P4333
Fetal bovine serum (FBS)	Capricorn Scientific GmbH	FBS-12A
Bovine serum albumin (BSA)	Sigma-Aldrich	A9418
Triton X-100	Merck Millipore	108603
EdU	Abcam	ab146186
Dimethyl sulfoxide (DMSO)	Sigma-Aldrich	W387509
Atto 488 azide	ATTO-TEC GmbH	AD 488-101
Copper (II) sulfate pentahydrate	Sigma-Aldrich	209198
L-Ascorbic acid	Sigma-Aldrich	A0278
Trizma base	Sigma-Aldrich	T6066
Paraformaldehyde	Sigma-Aldrich	158127
Agarose	Sigma-Aldrich	A9539
Dulbecco’s modified Eagle’s medium (DMEM)	Sigma-Aldrich	D6429
Dulbecco’s phosphate-buffered saline (PBS)	Sigma-Aldrich	D8537
Trypan blue stain 0.4%	Sigma-Aldrich	T8154
Trypsin-Ethylenediaminetetraaceticacid (EDTA)	Sigma-Aldrich	T3924
Ethanol absolute ≥99.5%, TechniSolv, pure	VWR	83813.360
Vectashield antifade mounting medium for fluorescence	Vector Laboratories	H-1000

**Critical commercial assays**		

MicroTissues 3D Petri dish micro-mold Tech evaluation kit	Sigma-Aldrich	Z764116-8EA

**Experimental models: cell lines**		

Human: MCF7 breast epithelial cells	ATCC	HTB-22

**Software and algorithms**		

ImageJ	Schneider et al., 2012	https://imagej.nih.gov/ij/
Leica Application Suite X (LAS X)	Leica Microsystems	https://www.leica-microsystems.com/products/microscope-software/p/leica-las-x-ls/

**Other**		

Petri dish, 100 × 20 mm	Greiner Bio-One	664160
6-well plate, PS, with lid	Greiner Bio-One	657102
Countess cell counting chamber slides	Invitrogen	C10228
Countess II automated cell counter	Invitrogen	AMQAX1000
Tube, 15 mL, PP, screw cap	Greiner Bio-One	118271
μ-Slide 8-well high glass bottom	ibidi	80807
1.5 mL microcentrifuge tube, low binding	VWR international B.V. (Biotix)	MTL-0150-BC

## Materials and equipment

**Table:** Spheroid culture medium

Reagent	Final concentration	Amount
DMEM	1x	500 mL
FBS	10%	50 mL
Penicillin-streptomycin	1%	5 mL
**Total**	**N/A**	**555 mL**

Store at 2°C–8°C for up to 1 month. Before use, warm the medium to 37°C in a water bath.

**Table:** Click-IT reaction mix

Reagent	Final concentration	Amount
Tris buffer (50 mM)	39.5 mM	1580 μL
Atto 488 Azide (6 mM)	60 μM	20 μL
Copper (II) sulfate pentahydrate (40 mM)	4 mM	200 μL
L-Ascorbic acid (100 mM)	10 mM	200 μL
**Total**	**N/A**	**2000 μL**

Use within 15 min of preparation. Keep on ice and protect from light.

## Step-by-step method details

### Casting of agarose in MicroTissues molds

**Timing: 1 h**

**Figure 1:**
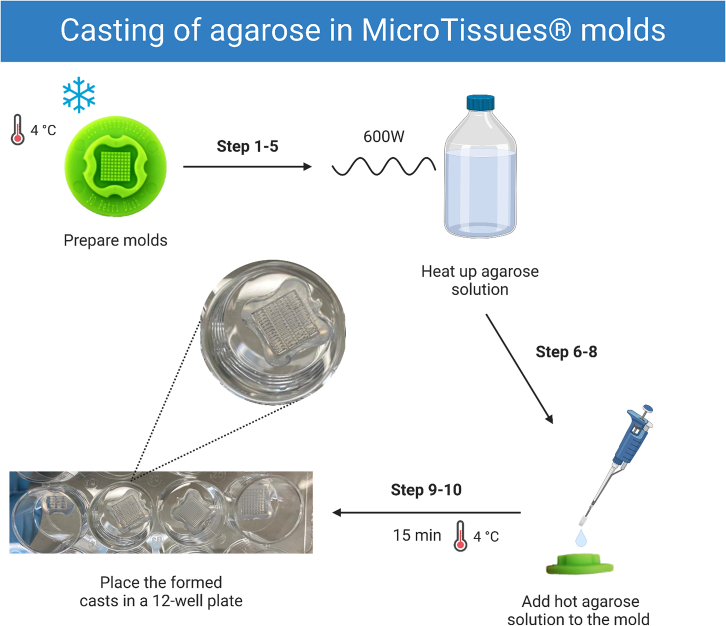
Workflow of making agarose casts with MicroTissues molds The images correspond to the indicated steps.

In this step we describe how to use MicroTissues molds to form agarose casts required for the formation of spheroids and rod-shaped micro-tissues. Spheroids can be formed in various sizes to suit the setup of the experiment. Rod-shaped micro-tissues are much larger than spheroids and can be useful to mimic the characteristic of a tissue slice including potential hypoxia and/or necrosis in the core region. Figure 1 gives a graphical overview of this step.

1. Use the correct molds needed for formation of spheroids or rods (Table 1).
2. Prepare molds for casting by rinsing with 70% (v/v) ethanol before use.
3. Air-dry molds on a paper towel in the biosafety cabinet.
4. When completely dry, place the molds in a 10 cm dish and let them cool at 4°C for 10–15 min.
5. For casting, prepare a sterilized 2% (w/v) agarose solution in phosphate-buffered saline (PBS).
6. Heat up the agarose solution using a microwave at 600 W (loose lid) until the agarose has been molten.

***Note:*** Make sure to stir the bottle every 10–15 s when heating up.

7. Let the agarose solution cool down to approximately 70°C before use.
8. Pipette 700 μL (12-well series) or 400 μL (24-well series) of agarose solution directly in the molds.

**CRITICAL:** Avoid air-bubbles when pipetting agarose, this may cause that a part of the mold is not properly filled with agarose.

9. Let agarose casts solidify at 4°C for 15 min.
10. When the agarose has solidified, push the cast out of the mold.
  a. Place the mold between your index fingers and thumbs.
  b. Start stretching the silicone material in the corners of the mold outward.
  c. When you can see that the cast is loose, turn the mold upside down and drop it carefully in a 12-well plate for further use.
  d. Verify proper cast formation under a light microscope.

***Note:*** Make sure that there are no cracks visible either on the edge or the middle of the cast.

***Note:*** Optionally, casts can be stored submerged in culture medium for up to 4 weeks at 37°C.

**CRITICAL:** Do not break or damage the agarose cast as any leakage will later on cause problems with spheroid or rod formation.

**Table 1:** Micro-mold information from MicroTissues

Mold type	Shape microtissues	Dimensions
12–81	Spheroids	Diameter: 800 μm, Depth: 800 μm
12–256	Spheroids	Diameter: 300 μm, Depth: 800 μm
24-24TR	Rods	Length: 2200 μm, Width: 400 μm, Depth: 800 μm
12-60TR	Rods	Length: 2200 μm, Width: 400 μm, Depth: 750 μm

### Cell seeding in casts for spheroid and rod formation

**Timing: 1–1.5 h**

**Figure 2:**
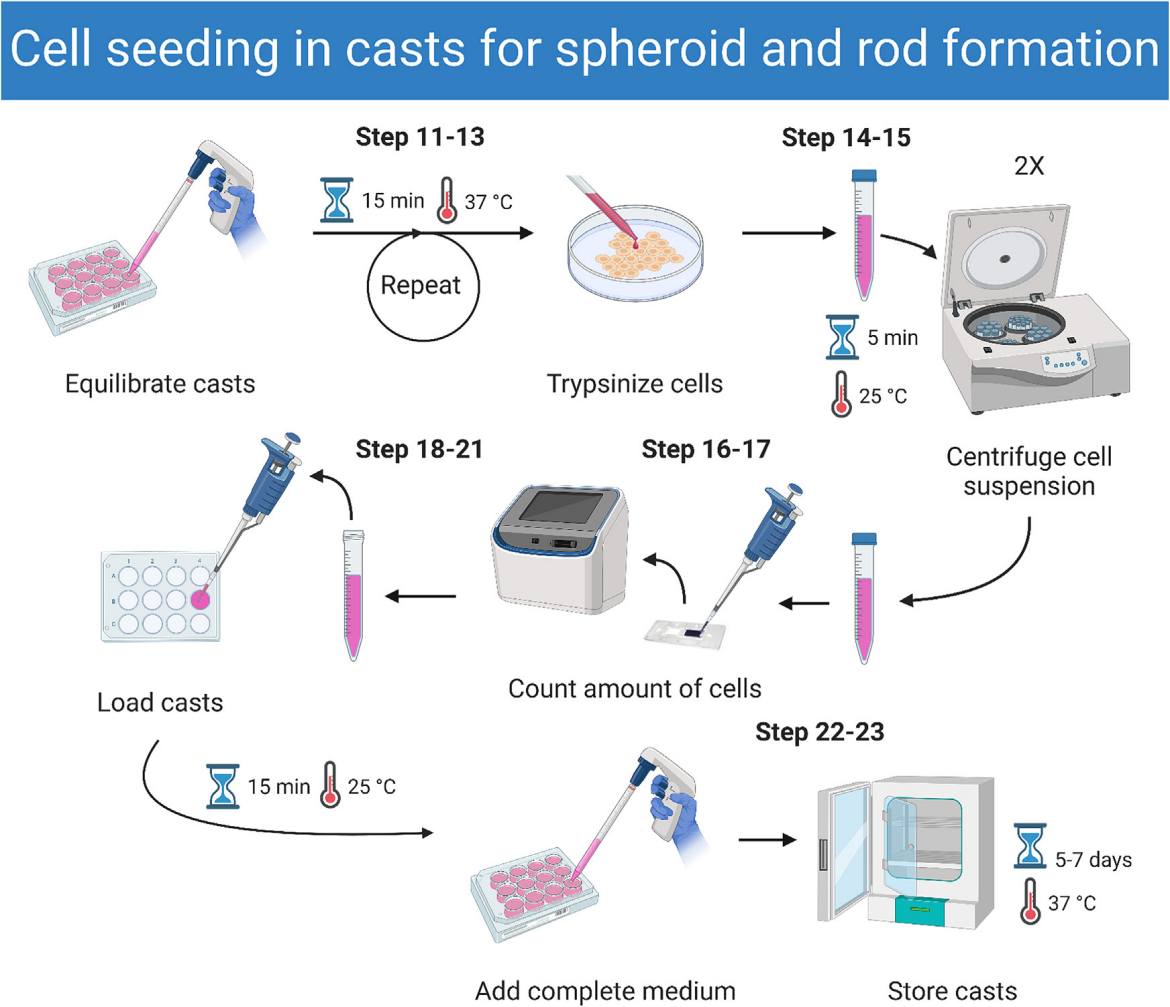
Workflow of seeding cells in casts for spheroid and rod formation The images correspond to the indicated steps.

**Figure 3:**
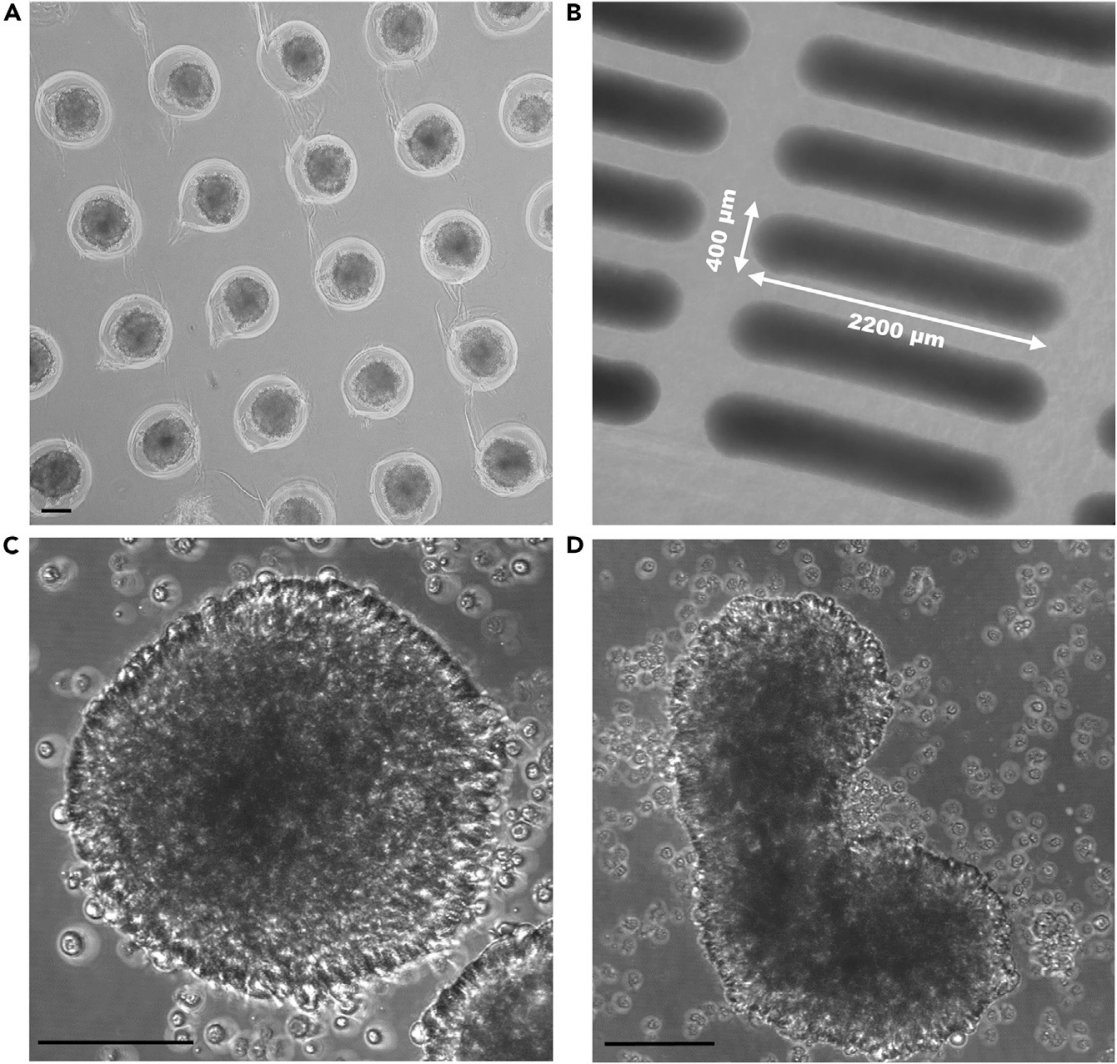
Spheroid and rod formation of MCF7 cells MCF7 spheroids in 12–256 agarose cast 7 days after seeding (A) and zoom-in (C). MCF7 cells seeded in 12–60TR to form rods (B) and zoom-in of rod-shaped micro-tissue (D). Images were taken using the Olympus IX70 microscope and SP8 TCS Confocal Microscope respectively. Scale bars represent 100 micron.

In this step we describe how to seed cells in the agarose casts that were made in the previous section. Figure 2 gives a graphical overview of this step and Figure 3 shows formed spheroids and rods.

11. Equilibrate casts two times with pre-warmed (37°C) 2.5 mL medium (DMEM supplemented with 10% FBS and 1% penicillin-streptomycin) at 37°C for 15 min.
12. Wash the cells once with pre-warmed 1x PBS.

***Note:*** It is recommended to prepare at least one 80%–90% confluent 100 × 20 mm culture dish with cells (containing around 5.0∗10^6^ cells) per cast to ensure sufficient cell numbers for seeding.

13. Incubate the cells with 0.05% Trypsin with 1 mM EDTA for at least 2–3 min at 37°C.
14. Re-suspend the cells in medium, transfer the cells to a 15 mL Falcon tube and centrifuge at 200 × *g* for 5 min at 20°C–22°C.
15. Discard supernatant, re-suspend cells in medium and repeat centrifugation.

**CRITICAL:** Centrifugation must be done twice as described to allow optimal spheroid and rod formation.

16. Remove supernatant and re-suspend cells in 1–2 mL of medium.

***Note:*** A greater volume can be used when lower cell concentrations are required.

17. Add 10 μL of cell suspension to an equal volume of 0.4% (v/v) Trypan blue solution and pipette 10 μL in both sides of a cell counting chamber slide.

***Note:*** Trypan blue solution will stain any dead cells present in the counted cell population. It is very important to use the live cell numbers for further calculations.

18. Count live cells with the Countess II automated cell counter and dilute cell suspension accordingly.
19. Discard all medium surrounding the casts and also in the cell seeding chamber.
20. Load 190 μL (12-well series) or 75 μL (24-well series) of cell suspension directly in the cell seeding chamber of the cast (Cell concentrations: Table 2).
21. Let cells settle in the micro-wells for a minimum of 15 min at 20°C–22°C.
22. Carefully add 2.5 mL pre-warmed medium to the edge of the well.

**CRITICAL:** Make sure not to pipette this volume directly into the cast, since this will disturb the settled cells in the cast.

23. Leave the cells in the incubator for 5–7 days at 37°C (5% CO_2_, 90%–95% humidity).

***Note:*** Optionally, the medium can be changed as soon as 3 days post-seeding.

**Table 2:** Cell seeding amounts for various cast types for MCF7 cells

Cast type	Cell concentration/mL	Total amount of cells loaded
12–81	5.0∗10^5^	9.50∗10^4^
12–256	1.2∗10^6^	2.28∗10^5^
24–24TR	5.0∗10^6^	3.75∗10^5^
12–60TR	5.0∗10^6^	9.50∗10^5^

### Treatment of cell models with DNA damage inducing agents

**Timing: 0.5 h**

This step is optional. In this step we describe how to treat 3D cancer models with DNA damage inducing agents, which can be used to study treatment response in these models. We applied irradiation but also other treatments may be applied at this step. In this step we used spheroids or rods that were left in the incubator for 5–7 days after cell seeding to form.

24. Irradiate spheroids or rods with 2 Gy of γ-irradiation 5–7 days after cell seeding.

***Note:*** The spheroids or rods were irradiated in the agarose cast and harvested 2 h after irradiation as described in the next section.

***Note:*** Irradiation with 2 Gy is an example of a treatment that induces DNA damage. Other treatments can also be used including drugs. In this case the drug needs to be directly added to the culture medium surrounding the cast.

### Spheroid and rod harvesting from agarose casts

**Timing: 2–2.5 h**

**Figure 4:**
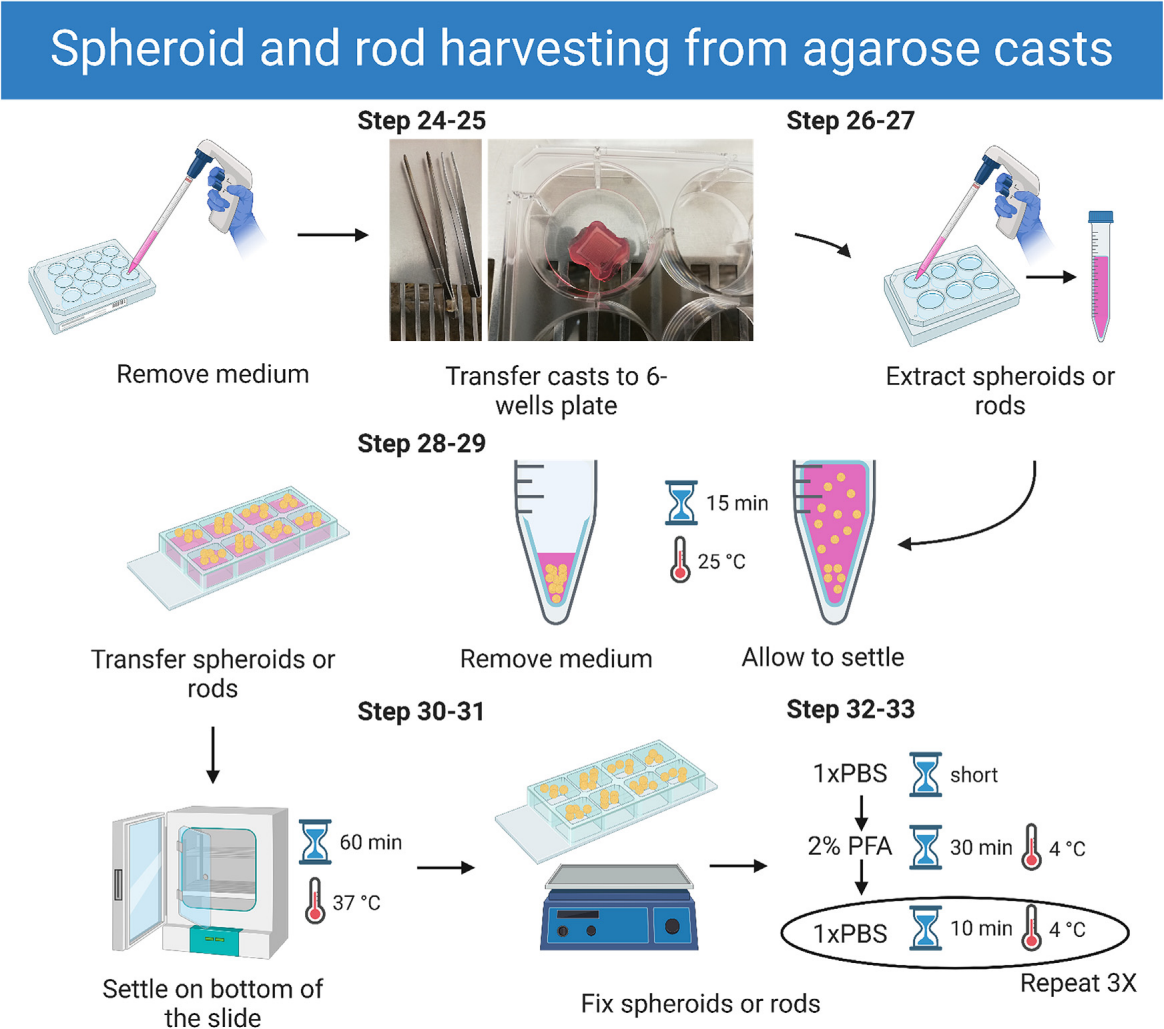
Workflow of spheroid and rod harvesting from agarose casts The images correspond to the indicated steps.

In this step we describe how to harvest formed spheroids and rods from the agarose casts to image them either directly or after fixation. Figure 4 gives a graphical overview of this step. If immunofluorescence staining will be performed on spheroids or rods, go directly to the next section.

25. Remove culture medium completely from the wells, both surrounding the casts and inside the cell seeding chamber.

**CRITICAL:** Make sure not to damage the casts as this will cause problems with harvesting or the overall yield respectively.

26. Carefully transfer each of the casts to a 6-well plate using sterile tweezers.
27. Extract spheroids or rods from the cast by pipetting 5 mL of culture medium directly onto the center of the cell seeding chamber.
28. Take up this volume and repeat a couple of times.

***Note:*** Verify that all spheroids or rods have been extracted from the cast under a light microscope.

**CRITICAL:** Do not pipet up and down too quickly as this will cause the spheroids or rods to fall apart.

29. Transfer suspension to a 15 mL centrifuge tube and let the spheroids or rods settle for a minimum of 15 min at 20°C–22°C.
30. Carefully remove the medium in the tube leaving spheroids or rods in circa 200 μL of medium.
31. Transfer the suspension to an 8-well μ-slide with glass bottom.

***Note:*** It is recommended to place the slide in a 10 cm dish for more stability and less disturbance of spheroids and rods when moving.

32. Let the spheroids or rods settle on the bottom of the μ-slide for approximately 1 h at 37°C.

***Note:*** For live-cell imaging, the μ-slide can be directly placed on the microscope table.

33. For imaging of fixed samples remove medium and wash spheroids or rods once with 150 μL 1x PBS, before fixation with 2% PFA for 30 min on a rocking platform at 4°C.
34. Remove PFA and wash spheroids 3 times for 10 min with 150 μL 1x PBS on a rocking platform at 4°C.
35. Discard all PBS and mount spheroids or rods in the μ-slide using 1 drop (circa 15–20 μL) of Vectashield mounting medium.

### Immunofluorescence staining of spheroids and rods

**Timing: 3–4 days**

In this optional step we describe how to perform immunofluorescence staining of spheroids and rods, combining EdU with antibody staining.

36. For EdU staining, incorporate spheroids with 3 μL of EdU for every mL of medium (final concentration: 30 μM) for 2 h at 37°C.
37. Harvest spheroids or rods as described in steps 25–28 of the previous section.
38. Transfer spheroids to a 1.5 mL low-binding Eppendorf tube and centrifuge at 100 × *g* for 5 min at 20°C–22°C.
39. Remove supernatant and add 1 mL of 1x PBS, centrifuge at 100 × *g* for 5 min at 20°C–22°C.
40. Remove PBS and fix spheroids with 1 mL 2% PFA for 30 min at 20°C–22°C on a rotation wheel.
41. Wash spheroids 3 times for 10 min with 1 mL 1x PBS at 20°C–22°C on a rotation wheel.
42. Centrifuge at 100 × *g* for 5 min at 20°C–22°C after every 10-min wash step.

***Note:*** Optionally, spheroids can be left overnight on 1x PBS at 4°C.

43. Centrifuge at 100 × *g* for 5 min at 20°C–22°C and remove PBS.
44. Add 1 mL blocking solution (1x PBS + 1% (v/v) Triton X-100 + 3% (m/v) BSA) and block and permeabilize spheroids for 1 h at 20°C–22°C on a rotation wheel.
45. Centrifuge at 100 × *g* for 5 min at 20°C–22°C, remove blocking solution and add 1 mL of Click-IT reaction mix.
46. Incubate for 2 h at 20°C–22°C in the dark on a rotation wheel.

**CRITICAL:** Make sure the Click-it mix is freshly made. If left for too long, the reaction does not work.

47. Centrifuge at 100 × *g* for 5 min at 20°C–22°C, remove Click-IT reaction mix and wash twice for 10 min with 1 mL blocking solution at 20°C–22°C on a rotation wheel.
48. Centrifuge at 100 × *g* for 5 min at 20°C–22°C after every 10-min wash step.

***Note:*** Protect spheroids from light.

49. Remove blocking solution and add 1 mL of new blocking solution. Block and permeabilize for 4 h at 20°C–22°C on a rotation wheel in the dark.
50. Centrifuge at 100 × *g* for 5 min at 20°C–22°C, remove blocking solution and add 1 mL of primary antibodies dilution in blocking solution.

***Note:*** The optimal antibody dilution should be determined and may differ depending on the antibody.

51. Incubate for 24–48 h at 4°C on a rotation wheel.

***Note:*** Incubation time and temperature might defer depending on the antibody.

52. Centrifuge at 100 × *g* for 5 min at 20°C–22°C, remove dilution of primary antibodies and wash three times for 10 min with 1 mL 1x PBS+0.1% (m/v) BSA at 20°C–22°C on a rotation wheel.
53. Centrifuge at 100 × *g* for 5 min at 20°C–22°C after every 10-min wash step.
54. Remove PBS and add 1 mL of the secondary antibody dilution in blocking solution.
55. Incubate for 3 h at 4°C on a rotation wheel.
56. Centrifuge at 100 × *g* for 5 min at 20°C–22°C, remove secondary antibodies dilution and wash three times for 10 min with 1 mL 1x PBS at 20°C–22°C on a rotation wheel.
57. Centrifuge at 100 × *g* for 5 min at 20°C–22°C after every 10-min wash step.
58. Remove PBS and mount spheroids in the tube with 15–20 μL of Vectashield mounting medium containing 4′,6-diamidino-2-phenylindole (DAPI) overnight at 4°C.
59. Transfer spheroids in mounting medium to a μ-slide for imaging.

**CRITICAL:** Protect spheroids from light to prevent fading of fluorophores.

### Confocal imaging of spheroids and rods

**Timing: 3 h**

In this step we describe how to image either fixed or live spheroids and rods using the Leica TCS SP8 confocal microscope. This step can also be performed using the Leica TCS SP5 confocal microscope, but laser power might defer. When using a different brand of microscope, it is best to follow the manual of the manufacturer.

60. Open the LAS X software to allow usage of the microscope.
61. Activate all needed lasers, by going to the *Laser Configuration tab.*

***Note:*** When activating the Argon laser, put the power slider on 20%.

62. Go to the *Acquire tab* and start adjusting the settings for the particular experiment.
  a. Activate the UV and Visible light lasers by switching from OFF to ON.
  b. Turn off all detectors using the OFF/ON button.
  c. Activate ‘bidirectional X’.
    ***Note:*** The activation allows bidirectional scanning.
  d. Activate the sequential scanning mode by pressing ‘Seq’
  e. Choose to image ‘between frames’.
    ***Note:*** It is not always needed to use sequential scanning as this is only important when imaging fluorophores with overlapping emission spectra.
  f. Add as much scans as needed by pressing ‘+’.
  g. Select the first scan and activate the desired detector using the OFF/ON button.
    ***Note:*** We used mainly photomultiplier (PMT) detectors, since the fluorescence intensity of our reporter plasmids was relatively high. When imaging low levels of fluorescence, switching from PMT to a Hybrid Detector (HyD) might help making better images as it is roughly 2–3 times more sensitive.
    ***Optional:*** When live-imaging is performed, it is possible to also activate the transmission image detector under the TLD window.
    ***Note:*** One of the PMT detectors in this scan has to be activated as well to make it work.
  h. Adjust the gain of the activated PMT detector to 800 V initially, make sure offset percentage is at 0.
  i. Adjust the pseudo color display for the activated detector.
  j. Set up proper gating for the fluorophore that you want to detect.
    ***Note:*** The gating should always be 10 nm away from the excitation wavelength of the used laser. It is also recommended to narrow the gates for a specific fluorophore if the emission spectrum overlaps or is close to another fluorophore that is imaged simultaneously. It can also be widened when the signal of the fluorophore is low.
  k. Set the specific laser power for this scan at 2% initially.
  l. Repeat for all other scans.
63. Select the 20X dry objective under the objective window.

***Note:*** This objective is used to maximize the working distance to allow imaging through as much cell layers as possible.

64. Place the μ-slide on the microscope stage and secure it with stage clips on both sides.
65. Change the Z-position of the objective until focus on spheroids or rods is achieved.
66. Select the first scan and press ‘Live’.
67. Change the Z-position by moving the microscope stage to reach the middle of the spheroid or rod.
68. Adjust settings based on the observed image.
  a. If the signal is overexposed, reduce the gain below 600 V. When still overexposed, reduce the gain further or lower the percentage of laser power.
  b. If the signal is underexposed, increase the laser power (3%–5%) or switch detectors from PMT to HyD.
    ***Note:*** Do not increase the gain above 800 V as this will create more background noise. Usually it is best to have the gain around 600 V or lower.
    ***Optional:*** See if the gain can be lowered. You can increase the laser power percentage (3%–5%) to compensate for this adjustment. This way, the best images with the least background are obtained. Make sure not to put the laser intensity too high as this can create photo-bleaching.
69. Adjust the settings of the other scans in the same manner as described.
70. Change the X/Y-position to place the spheroid or rod in the middle of the image.
  ***Optional:*** When imaging rods, put the zoom on 0.9 as this will allow imaging of the entire structure in a single field of view.
71. Go to the Z-stack window and change the settings for this particular spheroid or rod.
  a. Select the first scan and press ‘live’.
  b. Change the Z-position by moving the microscope stage to reach the top part of the spheroid or rod. Mark this point as ‘Begin’.
  c. Change the Z-position again by moving the microscope stage to reach to bottom part of the spheroid or rod. Mark this point as ‘End’.
    ***Note:*** When imaging live, it is likely that you will not be able to visualize the entire spheroid or rod completely as light scattering will get worse the further you will image into the structure. You can mark the point where you lose the signal as ‘End’ in that case.
  d. Click the ‘Nr. Of steps’ button and change this to the desired amount of imaging steps it takes to go from the beginning of the Z-stack to the end.
    ***Note:*** The amount of steps will result in a certain step size. The optimal step size depends on the type of cells and/or features that you want to analyze.
  e. Change the imaging format from 512 × 512 pixels to 1024 × 1024 or even 2048 × 2048 pixels depending on what is preferred.
    ***Note:*** The higher the imaging format, the longer it will take to acquire the image.
  f. Scan speed should be between 400 and 600 Hz.
    ***Note:*** If the scan speed is too low, it can increase photo-bleaching whereas too high scan speed will result in more background noise.
72. Press ‘start’ and wait for the imaging procedure to finish.
73. When done, find the next spheroid or rod that you want to image and change the Z-stack settings accordingly.
74. When imaging is complete, save all files with correct labeling. Image files can be opened later using LAS X software or ImageJ.

## Expected outcomes

In this protocol we describe the formation, treatment, harvesting, immunostaining and imaging of breast cancer spheroids and rod-shaped micro-tissues. To show different applications of the protocol we described the use of several different mold systems, two different breast cancer cell lines and cells with reporters. The use of the 12–256 and 12–81 mold system to form MCF7 and MDA-MB-436 spheroids resulted in spheroids 200–300 micron in size 5–7 days after cell seeding. With the 12–60TR system for MCF7 cells we obtained micro-tissues 7 days after cell seeding (Figure 3).

**Figure 5:**
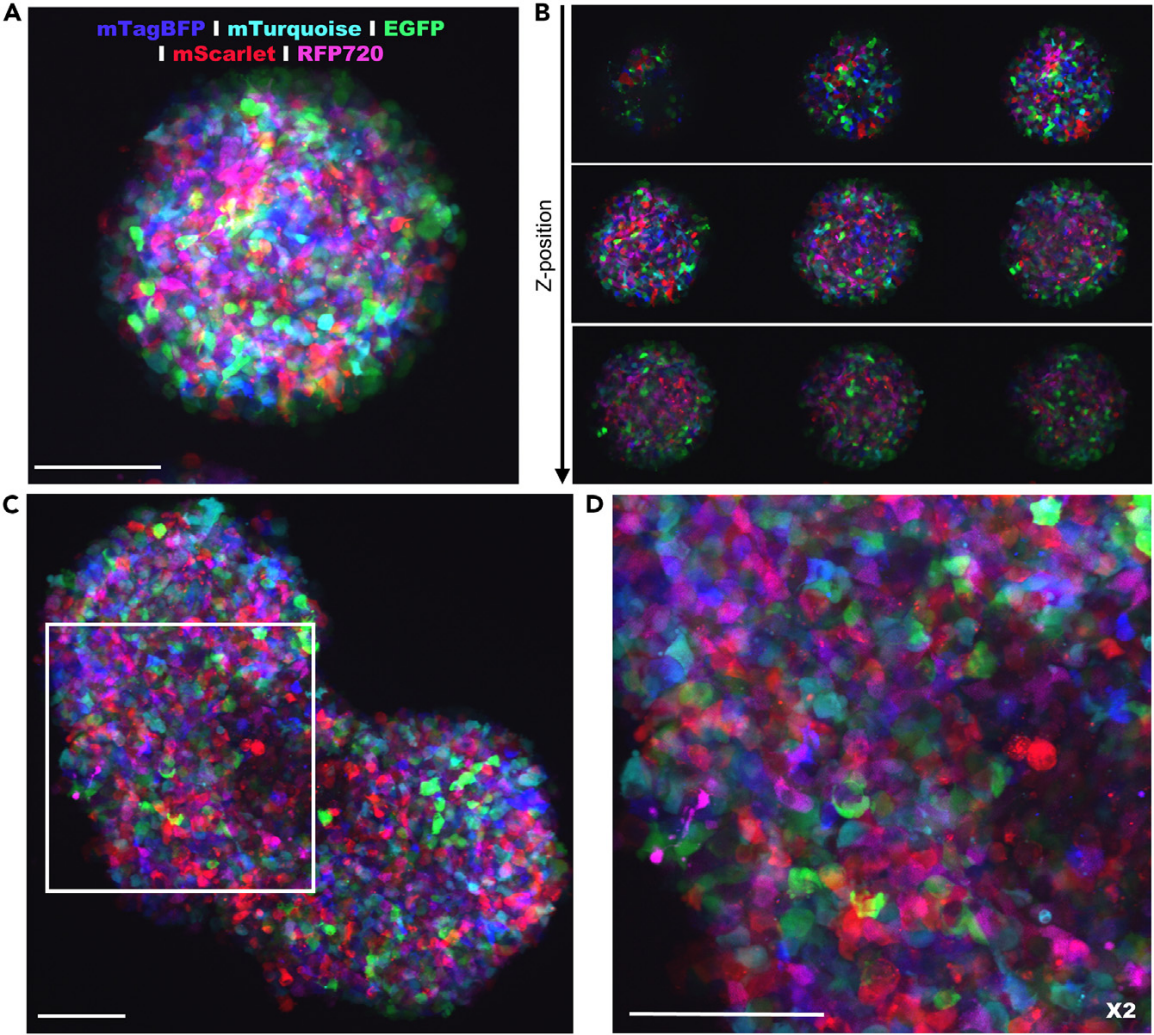
Confocal images of fixed MCF7 spheroid and rods Transfected MCF7 cells with mTagBFP (blue), mTurquoise (cyan), EGFP (green), mScarlet (red) and RFP720 (magenta) were mixed. Spheroids were made with the 12–256 mold system, fixed and imaged with the SP8 confocal microscope. The figure shows the maximum average projection (A) and z planes of the z-stack (B). Rods were made with the 12–60TR system shown completely (C) and with zoom-in (D). Scale bars represent 100 micron. Spheroids and rods were harvested for imaging 7 days after cell seeding.

**Figure 6:**
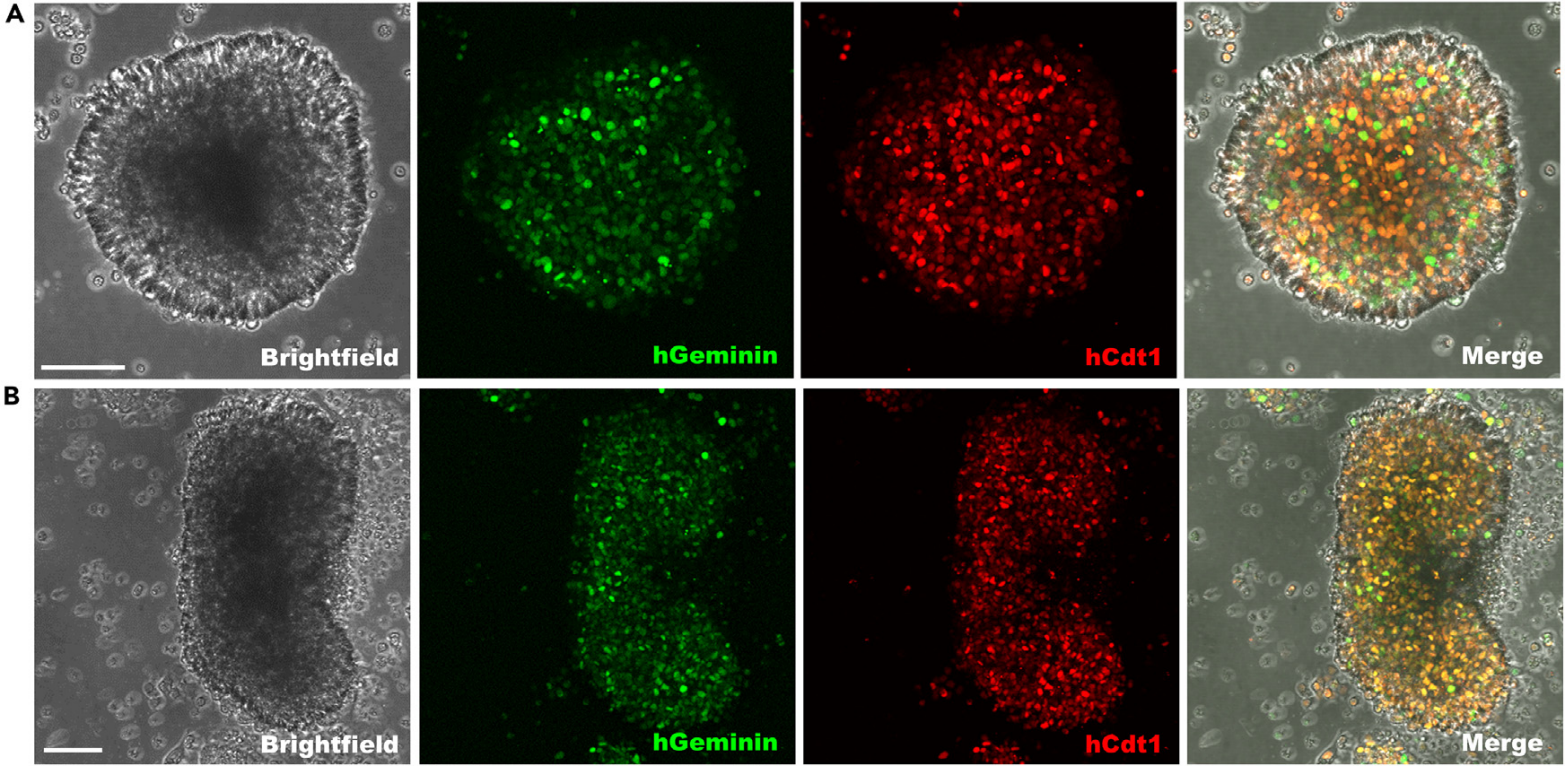
Confocal images of live MCF7 spheroid and rods with Fucci reporter Spheroids (A) and rods (B) with cells in the S/G2/M phase of the cell cycle are geminin positive (green) and in G1 phase are CDT1 positive (red). Spheroids were made with the 12–81 system and rods with the 12–60TR system. Scale bars represent 100 micron. Spheroids and rods were imaged 7 days after cell seeding.

The confocal imaging described in the protocol allows imaging of breast cancer cells with multiple colors. We show an example of imaging fixed spheroids and rods with MCF7 cells of five different colors (Figure 5). In Figure 6 we show live imaging of spheroids and rods to follow the cell cycle with cells containing the Fucci construct.^2^

**Figure 7:**
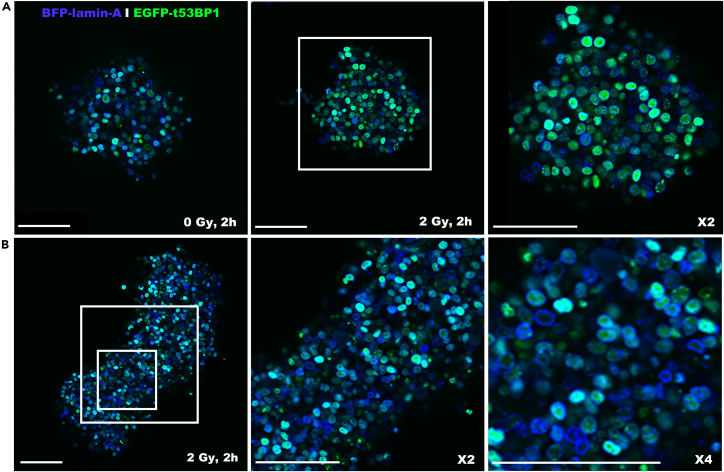
Imaging of irradiated MCF7 spheroids and rods with 53BP1-reporter Spheroid (A) and rod formation (B) of MCF7 cells, stably expressing EGFP-truncated 53BP1 (DNA damage) and nuclear marker BFP-laminA irradiated with 2 Gy and fixed 2 h later. Foci of 53BP1 indicate double strand DNA breaks in the nuclei. Scale bars represent 100 micron. Spheroids and rods were irradiated 7 days after cell seeding. This figure shows a single plane from a z-stack image.

**Figure 8:**
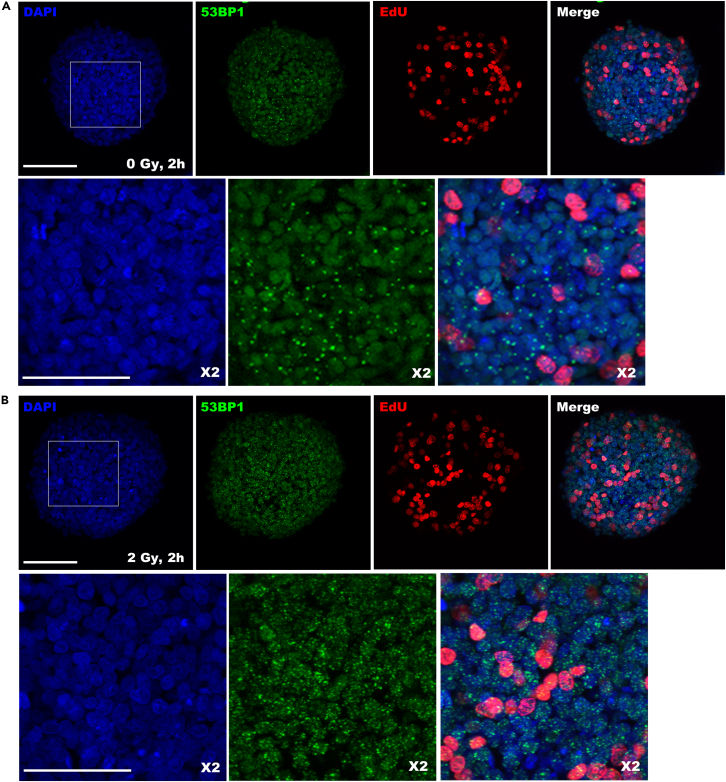
Whole-mount immunostaining of irradiated MCF7 spheroids Spheroids were harvested and fixed 2 h after irradiation (B) or mock-treatment (A) and stained for DNA double strand marker 53BP1 and cell proliferation marker EdU. Nuclei of cells were stained with DAPI. Scale bars represent 50 or 100 micron (zoom-in). Spheroids were irradiated 7 days after cell seeding. This figure shows the maximum average projection from a z-stack image.

Optionally, cells can be treated with DNA damage inducing agents such as irradiation. When spheroids or rods have been irradiated with 2 Gy of γ-irradiation, more 53BP1 foci are visible in the nuclei of cells indicating the induction of double strand DNA breaks upon irradiation (Figure 7). To study treatment effect in 3D cancer models, these can also be stained for DNA damage and cell proliferation markers. Our protocol describes the whole mount immunostaining of spheroids (which can also be applied to rods) for EdU to identify proliferating cells and an antibody staining for 53BP1 (DNA damage). Upon irradiation with 2 Gy more 53BP1 foci are visible in the MCF7 spheroids, indicating an increase in DNA double strand breaks due to the treatment (Figure 8).

**Figure 9:**
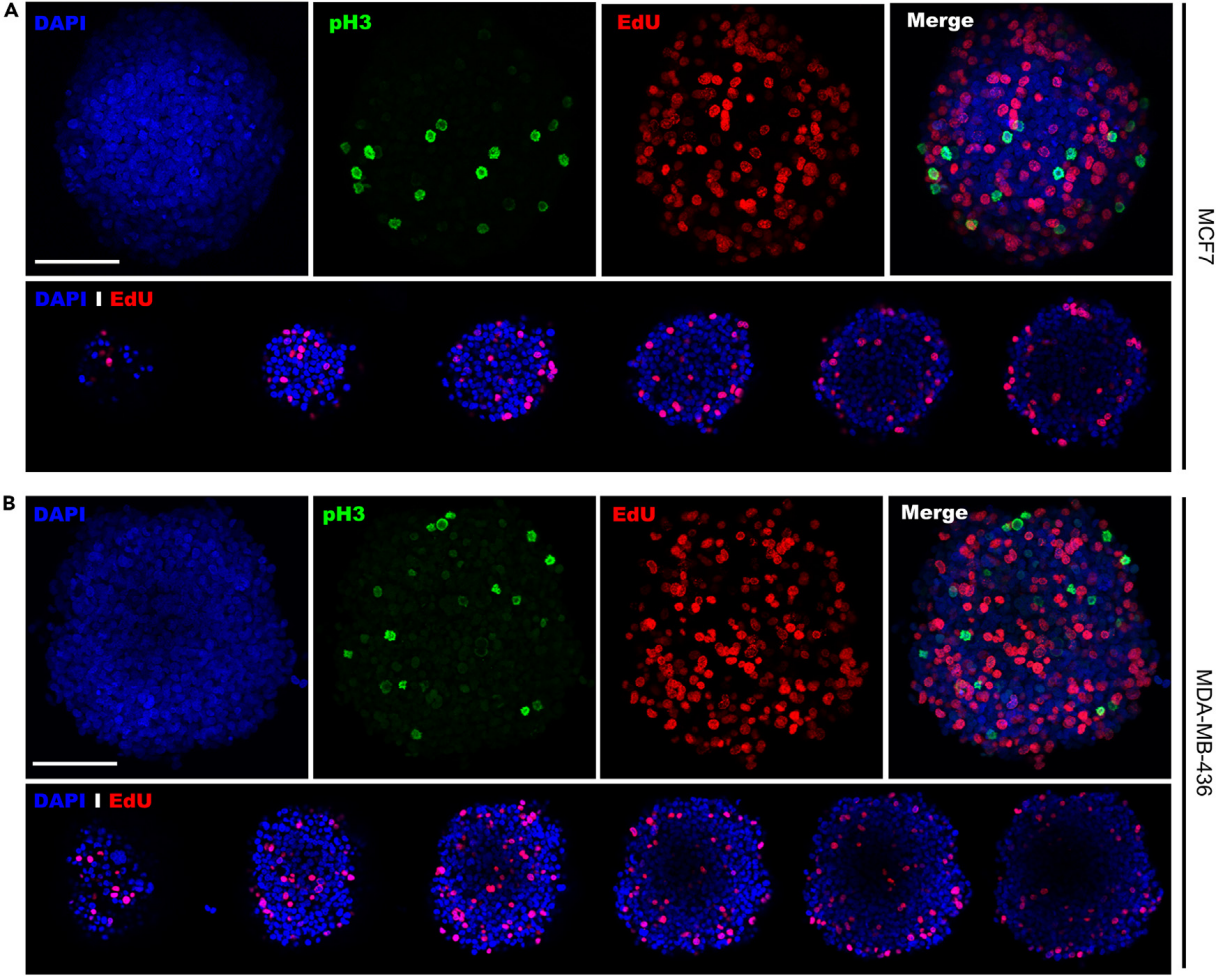
Whole-mount immunostaining of MCF7 and MDA-MB-436 spheroids stained for cell proliferation marker EdU and mitosis marker pH3 MCF7 (A) and MDA-MB-436 (B) spheroids were harvested and fixed 7 days after cell seeding and stained for cell proliferation marker EdU (red) and mitosis marker pH3 (green). Nuclei of cells were stained with DAPI. Scale bars represent 100 micron. This figure shows the maximum average projection from a z-stack image.

Lastly, we stained spheroids of both breast cancer MCF7 and MDA-MB-436 models with EdU and pH3 as markers for cell proliferation and mitosis respectively. Proliferating cells are present throughout the whole spheroid in both models, observed by the high number of EdU-positive cells, throughout the spheroid whereas hardly any pH3 cells are present, suggesting only few cells are in mitosis (Figure 9).

## Limitations

This protocol works well for MCF7, MDA-MB-436, A549 and DU145 cell lines. However, for every new cell line the spheroid and rod formation must be tested and may require optimization as some cell lines need more extracellular matrix to form 3D structures. To overcome this limitation, a drop of extracellular matrix (such as Matrigel or fibronectin) may be added to the medium after loading the cell suspension into the wells to increase the chance of proper spheroid or rod formation. Also, the success rate of this protocol greatly depends on how well the initial formation of agarose casts is performed. Damaged casts may cause problems with ‘cell loading’, which will result in smaller or uneven 3D models. To overcome this limitation, great care has to be taken to avoid damage of the agarose casts. Lastly, 3D imaging is hampered by the working distance of the used microscope as well as light scattering that occurs when imaging through multiple cell layers. To overcome this limitation as much as possible, the most suitable combination of fluorophores, microscope and objective must be used to image the 3D structures.

## Troubleshooting

### Problem 1

Low quality of formed agarose casts including bubbles, damaged wells, cracks (related to steps 7–10).

### Potential solution

Make sure not to pipette any air-bubbles into the mold when adding the molten agarose (step 8). Secondly, proper formation of the casts is also very dependent on the temperature of the agarose when adding it to the mold. A lower temperature may make it difficult to properly fill the mold (step 7). Thirdly, the time for the agarose to solidify must not be cut short as the casts will crack more easily when taking them out of the mold (step 9). Lastly, it is recommended to practice a couple of times with making agarose casts as taking the casts out of the mold is a gentle procedure (step 10).

### Problem 2

Leakage of formed agarose casts when loading the cell suspension (related to step 10).

### Potential solution

It is likely that the agarose cast was damaged in the process (step 10). You will have to form this cast again as you will not be able to load the cells properly. This will cause uneven or smaller 3D cell models.

### Problem 3

No 3D cell models have formed after 7 days (related to steps 18–22).

### Potential solution

When counting the cells before loading, make sure to check the cell viability as low viability generally leads to less and smaller 3D cell model formation (step 18). It is very important to allow cells to settle in the agarose cast for at least 15 min as described (step 21). Otherwise cells will not be properly loaded in the micro-wells of the cast preventing proper formation of 3D cell models. Also, the pipetting of additional medium after loading the cells in the casts should not be done directly on the cast. Add the medium as slowly as possible to the edge of the well (step 22).

### Problem 4

Low yield of 3D cell models after harvesting (related to steps 25–32).

### Potential solution

When removing the medium from the casts it is recommended to do this slowly, as you may already take up 3D cell models and mistakenly throw them away (step 25). Secondly, harvesting must also be done gently thereby preventing the 3D structures from falling apart (step 27). Thirdly, this protocol includes optional washing and fixation steps. All these steps should be performed as gently as possible preventing further loss of 3D cell models and lowering overall yields (step 32). A yield of 80%–90% per cast should be achieved.

## Resource availability

### Lead contact

Further information and requests for resources and reagents should be directed to and will be fulfilled by the lead contact, Maayke M.P. Kuijten (m.kuijten@erasmusmc.nl).

### Technical contact

Technical questions should be directed to the technical contact, Ben Haspels (b.haspels@erasmusmc.nl).

### Materials availability

This study did not generate any new unique reagents.

### Data and code availability

The published article includes all generated data from this study.
